# Effectiveness of Dry Needling in Improving Pain and Function in Comparison with Other Techniques in Patients with Chronic Neck Pain: A Systematic Review and Meta-Analysis

**DOI:** 10.1155/2023/1523834

**Published:** 2023-08-23

**Authors:** Mar Hernández-Secorún, Hugo Abenia-Benedí, Sergio Borrella-Andrés, Isabel Marqués-García, María Orosia Lucha-López, Pablo Herrero, Isabel Iguacel, José Miguel Tricás-Moreno, César Hidalgo-García

**Affiliations:** ^1^Department of Physiatry and Nursing, Faculty of Sciences, University of Zaragoza, Zaragoza, Spain; ^2^Physiotherapy Research Unit, University of Zaragoza, Zaragoza 50009, Spain; ^3^Corpore 360°, Zaragoza 50008, Spain; ^4^iHealthy Research Group, ISS Aragón, University de Zaragoza, Zaragoza 50009, Spain

## Abstract

The purpose of this systematic review and meta-analysis was to assess the short-, mid-, and long-term effectiveness of dry needling in improving pain and functional capacity of patients with chronic neck pain. Search strategy was performed on PubMed, Web of Science, Scopus, PEDro, and Cochrane Library Plus biomedical databases. The risk of bias was assessed using the RoB2 tool. Randomised controlled clinical trials in which at least 1 of the groups received dry needling were included. 662 studies were found; 14 clinical trials were selected for qualitative analysis and 13 for quantitative analysis. The quality of most of the studies included was “high.” All the studies reported improvements in cervical pain and/or disability, regardless of the protocol followed and the muscles targeted. No serious adverse effects were reported. Dry needling showed to be more effective when compared with other therapies in both women and men, without differences by sex. When the analysis was carried out by age, patients over 40 years old benefitted more than those below 40 years old. Our meta-analysis supports the use of dry needling to improve pain and functional capacity in patients with chronic neck pain at short- and mid-term intervals.

## 1. Introduction

Neck pain is suffered by at least 30% of adults worldwide with a prevalence of 24439 to 61512 cases per 100000 population [1, 2]. Chronic symptoms are developed by 44% of the patients [3], and this condition is as important as lumbar pain in prevalence and duration [4]. When the problem turns chronic, there is an elevated economic and healthcare cost [5, 6].

Myofascial pain syndrome is defined as a set of autonomic, motor, and sensory signs and symptoms provoked by myofascial trigger points (MTPs) [7]. It often contributes to the appearance of mechanical neck pain [8] and it is associated with the chronification of the symptoms. A MTP is defined as a hyperirritable area in a skeletal muscle associated with a hypersensitive palpable nodule located in a taut band of muscle fibres [7]. The area is painful when subjected to mechanical deformation through compression, stretching, muscle contraction, or other stimuli; it can cause referred pain, hypersensitivity, motor dysfunction, and autonomic phenomena [7–10].

Different treatment strategies have been proposed to manage MTPs, being dry needling (DN) one of the most used [11]. The DN procedure consists of inserting a filiform, solid, nonbevelled needle into the MTP, without injecting or extracting any substance. DN is known to have a mechanical effect, provoking the disruption of dysfunctional motor endplates, and it is used to treat different pathologies [9]. DN has demonstrated to be effective in reducing myofascial pain in the upper [12] and lower quarter [13] in the short term. Moreover, DN has shown to be an effective and useful procedure complementary to conventional physiotherapy [14], either alone or in combination with pharmacological treatments [15] for headache management. In the case of neck pain, the current scientific evidence suggests that DN can be effective, although only in the short term [16]. Seventeen systematic reviews were published in relation to patients with neck pain and DN effectiveness. However, in the case of chronic neck pain, there are no reviews that have assessed the effectiveness of this technique. Moreover, sex and age characteristics are not usually considered when studying the effects of DN. Therefore, the objective of this systematic review and meta-analysis was to assess the short-, mid-, and long-term effectiveness of DN to improve chronic neck pain and functional capacity in comparison with other physiotherapy techniques or placebo. Secondary, the effectiveness of DN by subgroups, based on sex and age characteristics, was assessed.

## 2. Materials and Methods

This systematic review was conducted according to the PRISMA statement [17], designed and published to improve systematic reviews and meta-analyses. This review was registered on the Open Science Framework Registry digital platform: DOI 10.17605/OSF.IO/U6QRZ (https://osf.io/ywjbp). Abstract and PRISMA 2020 checklist can be found in Figures S1 and S2.

The PubMed, Web of Science, Scopus, PEDro, and Cochrane Library Plus electronic databases were included. In addition, a search of the grey literature was carried out (Google Scholar and ResearchGate). The search was performed from 15th September to 23rd December, 2021.

Our search strategy was established according to the recommendations of the Cochrane Back and Neck Group [18]. In agreement with these recommendations, three search categories were established (which were combined later) as follows: The purpose of the first category was to perform a sensitive search for the type of studies to be included: randomised controlled clinical trials or controlled clinical trials. The second category was designed to carry out a specific search for the condition of cervicalgia (neck pain or cervical pain). The purpose of the third category was to search specifically for the intervention of DN. See Figure S3 in the Supplementary Materials for Search Strategy. Search terms were established after a preliminary literature search, identifying the keywords and MeSH terms search. To identify additional registers, the search process ended with in-depth review of the bibliographic references included in the articles that underwent full text review.

Our systematic review included randomised controlled clinical trials in which at least 1 of the groups received DN as a treatment for chronic neck pain. The specific inclusion criteria included the following: (1) adult population (>18 years old); (2) chronic neck pain (>3 months); (3) superficial or deep DN technique; (4) description of the DN technique applied; (5) primary variables that included the intensity of the pain; the functional capacity or pain sensitivity (measured with pressure pain threshold); (6) articles written in English, Italian, French or Spanish languages. The exclusion criteria were as follows: (1) patients with neurological pain; (2) patients presenting headaches (tension-type headache, migraine or cervicogenic headache); (3) studies in which acupuncture was performed or mentioned as an intervention technique; (4) postoperative neck pain; and (5) studies published before 2010.

The articles extracted from each database were reviewed independently by two authors (M.H.S. and H.A.B.). Duplicate articles were eliminated using Covidence software. Selection of articles was carried out in three different steps: by title, abstract, and full text. Two independent reviewers (M.H.S. and H.A.B.) performed this selection and if a consensus was not reached, a third reviewer (S.B.A.) decided whether to include the article or not. Cohen's kappa index was calculated to assess the interrater agreement between the two primary reviewers [19].

The data on the studies selected were extracted by the two independent authors (M.H.S. and H.A.B.), filling in a standardised register excel sheet. The study characteristics recorded included the number of participants, the muscles on which the intervention was applied, the parameters used in the DN application, outcomes measured, and results achieved.

Both reviewers assessed the methodological quality and risk of bias independently. Methodological quality was evaluated using the scale of the Physiotherapy Evidence Database (PEDro) [20]. 11 items were assessed, giving each one a score from 1 to 0 depending on whether the item was fulfilled in the study or not, respectively. This scale establishes external validity using Item 1, internal validity using the items from 2 to 9, and result interpretability using Items 10 and 11. The first item was not taken into account in the final score, and 10 points was the maximum obtainable in this scale. Each article was classified according to the score obtained in the following manner: «high quality» if the score was greater than or equal to 6, «moderate quality» if the score was 4-5, and «low quality» if its score is less than 4.

The risk of bias 2 tool (RoB2) is the second version of the Cochrane tool to assess the risk of bias in clinical trials. The biases are evaluated in 5 domains: (1) randomization process; (2) effect of being assigned to intervention; (3) missing outcome data; (4) measurement of the outcome; and (5) reported results. Within each domain, 1 or more questions must be answered. These answers lead to the judgements of “low risk of bias,” “some concerns,” or “high risk of bias” [21].

All analyses were performed using RevMan Manager 5.4 software (The Cochrane Collaboration, 2012). The sample size, means, and standard deviation for each outcome were extracted. The mean difference (MD) with a 95% confidence interval (CI) was calculated for continuous data. In the cases, where different tools were used to assess pain or function, standard mean difference (SMD) was chosen. Sources of heterogeneity were investigated by subgroup analyses comparing results based on age (<40 years old, >40 years old. or NR, not reported); sex (mainly female, mainly men, and NR, not reported); and intervention (DN vs other intervention, DN vs DN + physical therapy (PT), and DN + PT vs PT). The heterogeneity of the studies was tested using the I^2^ statistic. This statistic describes the variance between studies as a proportion of the total variance. A value <25% indicated low heterogeneity, from 25 to 50% moderate, from 50 to 75% high heterogeneity, and >75% very high heterogeneity [22]. Funnels plots were performed for pain and function outcomes to explore any publication bias. In addition, a graphic display of heterogeneity (GOSH) was used, which plots the pooled effect size on the *x*-axis and the between-study heterogeneity on the *y*-axis, which allows looking for specific patterns or clusters with different effect sizes and amounts of heterogeneity (see Supplementary Materials, Figures S4–S6).

## 3. Results

### 3.1. Study Selection

**Figure 1:**
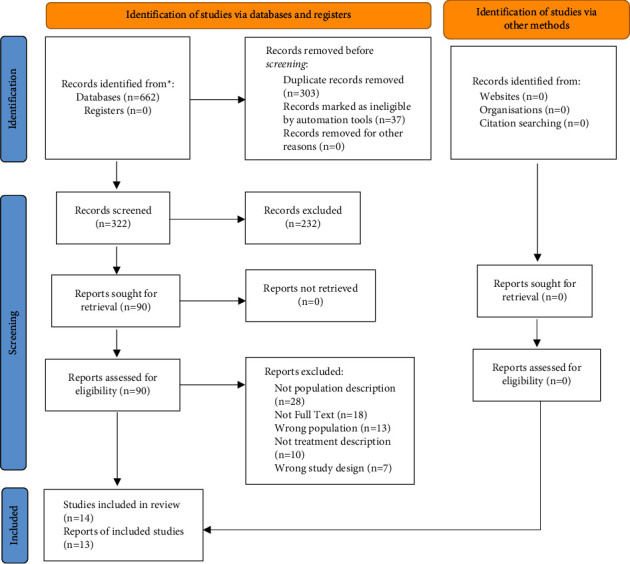
Flow diagram.

The search and the selection process of the relevant studies are shown in Figure 1. After the initial literature search, 662 studies were obtained. After eliminating the duplicated articles, the total number of articles left was 322. Of these, 232 studies were excluded based on the analysis of the title and summary/abstract. Finally, 14 studies were selected for the qualitative analysis and 13 for quantitative analysis. The kappa index between each author was 0.81 (95% CI: 0.65–0.91) [22].

### 3.2. Characteristics of the Studies

The studies characteristics are presented in Table 1. The DN technique was performed in the posterior cervical area (only one study did not specify the musculature involved) in all studies (22–35). The upper trapezius muscle was treated in 8 studies [23, 26, 27, 29–31, 33–35], levator scapulae in 5 studies [23, 25–27, 33, 35], the splenius and multifidus in 3 studies [25, 34, 35], and medium and lower trapezius in 3 studies [27, 32, 34].

The methodology of the technique application was not homogeneous, as there were variations regarding the number of local twitch responses produced, the duration of DN application, and the number of needle manipulations.

## 4. Effectiveness for Pain and Function

At short term (immediately after treatment—1 month), DN was more effective to decrease pain in 9 of the studies. In those studies, DN was compared with stretching (*p* < 0.001; 0.006) [25, 31], manual therapy (*p* < 0.001) [34], myofascial release (MR) (*p* < 0.001) [33], and electrotherapy using transcutaneous electrical nerve stimulation (TENS) with ultrasound (US) (*p* = 0.023) [24]. However, DN did not show statistically significant differences compared to extracorporeal shock wave therapy (ESWT) (*p* = 0.856) [30]. DN technique did not show any difference when percutaneous electrical nerve stimulation (PENS) (*p* = 0.504) [29], education (*p* > 0.05) [35], and manual therapy (*p* > 0.05) [23] were added. Moreover, DN showed to be more effective than miniscalpel-needle (MNS) (*p* < 0.001) [36]. As for the functional capacity, DN showed better results than stretching (*p* < 0.05) [31].

At mid term (1–3 months), both pain and functional capacity showed better results in the DN groups in all studies, except for the study of Stieven et al. that only showed improvements in the case of pain outcome. However, this was not the case when DN was compared with the miniscalpel-needle, in favour of the last one (*p* < 0.001) [36]. Moreover, no differences were found in the functional capacity when DN was compared with stretching (*p* > 0.05) [25]. In fact, worse results were found comparing DN alone versus DN combined with pain education [35], manual therapy (*p* > 0.05) [23], or PENS (*p* > 0.05) [28, 29]. In the case of pain, a better evolution was seen when DN was compared with stretching techniques (*p* < 0.05) [23].

At long term (>3 months), the results were contradictory. On the one hand, DN showed statistically significant improvements in pain reduction and functional capacity in all studies except for the one performed by Stieven et al. [34], which did not report significant improvements of DN versus MT combined with exercise (*p* = 0.13). On the other hand, statistically significant differences were found in favour of other treatments, such as MNS (*p* < 0.001) [36], MT (*p* < 0.001) [23], and PENS (*p* < 0.05) [28, 29], when it was compared to DN.

In the analysis of secondary variables, there was an improvement in the pressure pain threshold in the short- and mid-term intervals in all the studies in which this was measured [23, 25–33].

## 5. Methodological Quality

The mean score of the studies was 8.7, with 13 of the 14 selected studies having a high methodological quality and only one having a moderate quality. Therapist blinding was not achieved in any of the studies, while patient blinding was found in only 4 studies [24, 26, 27, 32]. Regarding the evaluator blinding, all studies had a blinded evaluator except one of them [27]. The details of the methodological quality scores of the articles assessed according to the PEDro scale can be found in Table 2.

**Figure 2:**
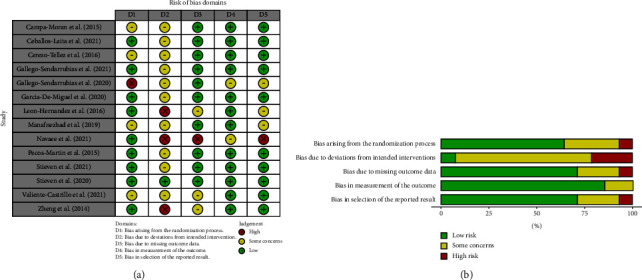
(a) Summary of risk of bias 2.0. (b) Risk of bias 2.0. graph.

The RoB2 tool shows that the features with the worst methodological quality were biased due to deviations from intended intervention, with approximately 25% being high risk. Bias in the measurement of the outcome was the domain with the best methodological quality in the set of studies, being more than 75%. The details regarding the risk of bias are presented in Figure 2.

## 6. Pain Meta-Analysis

**Figure 3:**
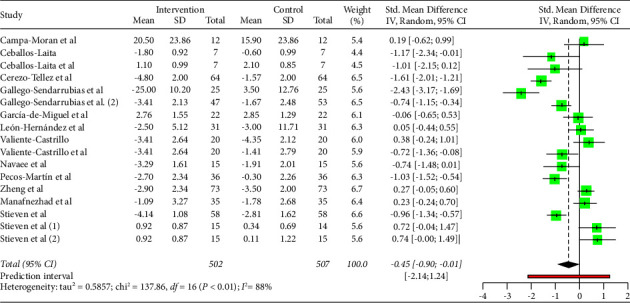
Pain analysis.

As shown in Figure 3, DN is effective to improve pain (MD: −0.45; 95% CI: −0.90; −0.01). However, heterogeneity was very high for the overall of studies (*I*^2^ = 88%; *p* < 0.01).

**Figure 4:**
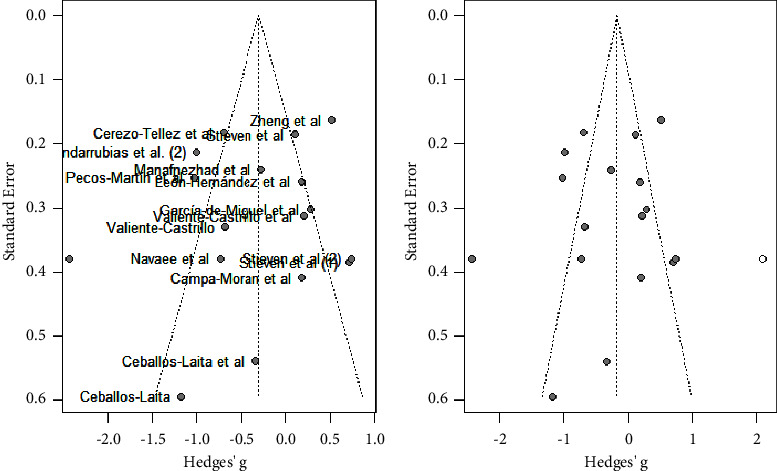
Pain analysis funnel plot.

As shown in Figure 4, the majority of studies followed a symmetrical distribution. So, it could be that the studies included in the analysis had no publication bias. In addition, the effect size was high for the majority of studies.

### 6.1. Subgroup Sex (Pain)

A subgroup analysis by sex was also carried out. No significant effects on pain were observed in the studies including mainly men (MD: −0.490; 95% CI: −1.713; 0.733) or mainly women (MD: −3.122; 95% CI: −5.309; 0.936). Only one study did not report the sex of the population. In this study, a significant effect on pain for the DN technique was not observed (MD: −1.380; 95% CI: −2.686; 0.074).

### 6.2. Subgroup Age (Pain)

**Figure 5:**
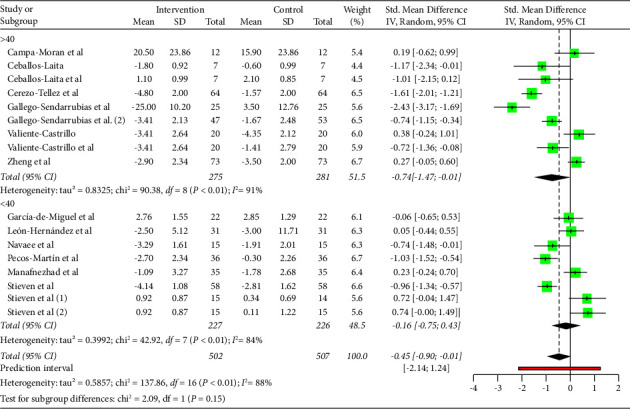
Pain subgroup analysis by mean age (<40 years old, >40 years old).

A subgroup analysis by age was performed, showing that DN was effective to improve pain in the studies in which the mean age was over 40 years old (MD: −0.74; 95% CI -1.47; −0.01). Nevertheless, no significant effects on pain were observed in the studies where the mean age was under 40 years old (MD: −0.16; 95% CI: −0.75; 0.43). Results are shown in Figure 5. Heterogeneity was very high and significant for studies in which mean age was over 40 years old (*I*^2^ = 91%) and high for those with a mean age under 40 years old (*I*^2^ = 84%).

**Figure 6:**
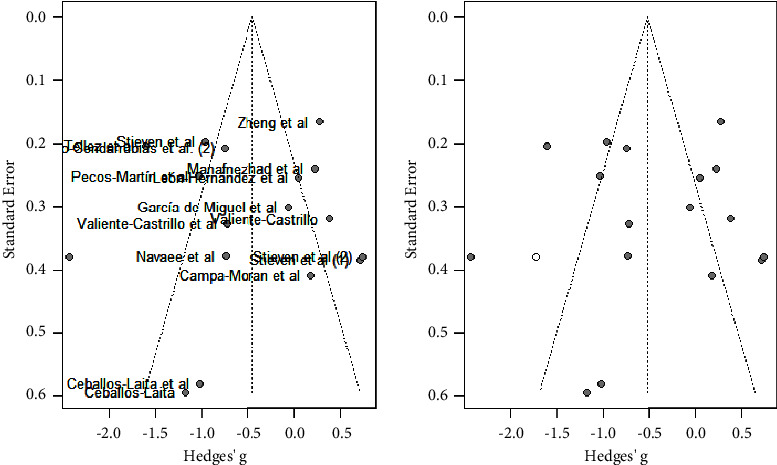
Pain subgroup analysis by mean age (<40 years old, >40 years old). Funnels plot.

As shown in Figure 6, the majority of studies did not follow a symmetrical distribution as shown in the funnel plot.

### 6.3. Subgroup Interventions (Pain)

**Figure 7:**
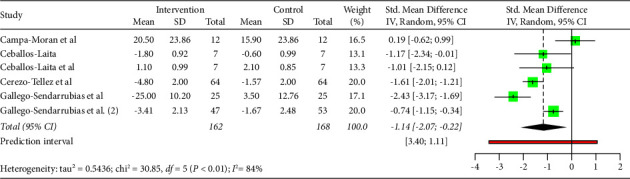
Pain subgroup analysis by intervention (DN + PT vs PT).

As shown in Figure 7, DN combined with physical therapy (PT) significantly reduced pain compared to physical therapy alone (MD: −1.14; 95% CI: −2.07; −0.22). Nevertheless, no significant differences were shown for DN alone compared to DN + PT (MD: 0.173; 95% CI: −0.549; 0.895) and DN compared to other interventions (MD: −1.236; 95% CI: −2.897; 0.425). Heterogeneity was very high and significant for studies comparing DN + PT vs PT (*I*^2^ = 84%; *p* < 0.01).

**Figure 8:**
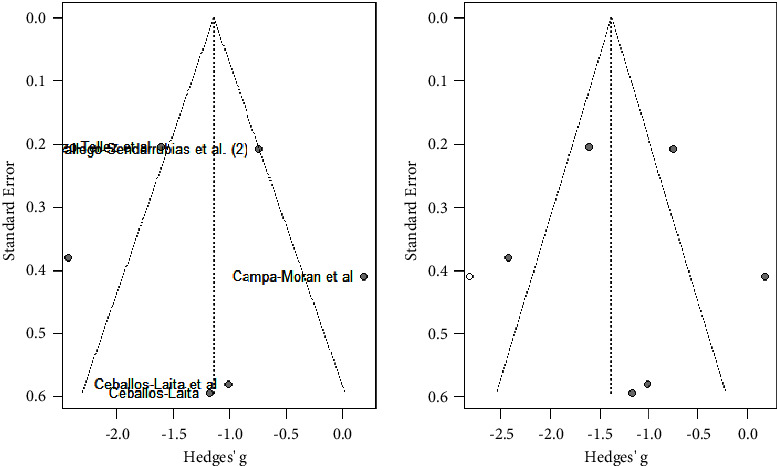
Pain subgroup analysis by intervention (DN + PT vs PT). Funnels plot.

As shown in Figure 8, all studies did not follow a symmetric distribution as shown in the funnel plot. So, probably, the studies included in the analysis had publication bias.

## 7. Function Meta-Analysis

**Figure 9:**
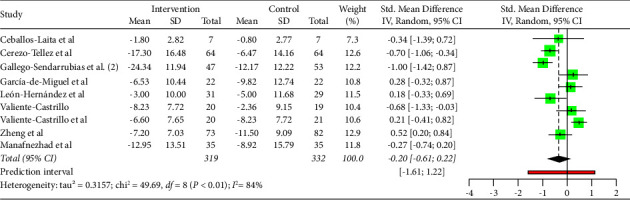
Function analysis.

As shown in Figure 9, DN was not statistically significant associated with improvements in function (MD: −0.20; 95% CI: −0.51; 0.22). Moreover, heterogeneity was very high for the overall of studies (*I*^2^ = 84%; *p* < 0.01).

**Figure 10:**
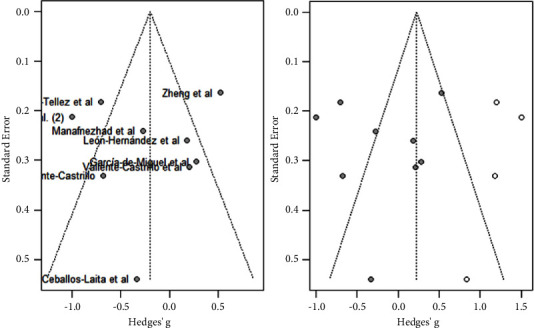
Function analysis funnels plot.

As shown in Figure 10, the majority of studies did not follow a symmetrical distribution. So, it could be that the studies included in the analysis had publication or information bias. The effect size was high for the majority of studies.

### 7.1. Subgroup Sex (Function)

A subgroup analysis by sex was carried out. DN was not significantly associated with improvements on function in studies in which the population was mainly females (MD: −1.701; 95% CI: −3.492; 6.894). Moreover, no significant effects on function were observed in the studies including mainly males (MD: −3.875; 95% CI: −8.058; 0.308). Heterogeneity was high for studies including mainly females (*I*^2^ = 86.07%) and for studies including mainly males (*I*^2^ = 78.42%).

### 7.2. Subgroup Age (Function)

Regarding the subgroup analysis by age, DN was not significantly associated with improvements on function in studies where the mean age was over 40 years old (MD: −2.299; 95% CI: −6.611; 2.013). Additionally, no significant effects on function were observed in the studies where the mean age was under 40 years old (MD: −2.897; 95% CI: −10.611; 4.817). Heterogeneity was high for studies in which mean age was over 40 years old (*I*^2^ = 85.53%) and for those with a mean age under 40 years old (*I*^2^ = 85.14%).

### 7.3. Subgroup Interventions (Function)

**Figure 11:**
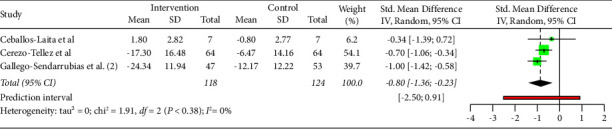
Function subgroup analysis by intervention (DN + PT vs PT).

As shown in Figure 11, DN combined with physical therapy (PT) significantly improved function compared to physical therapy alone (MD: −0.80; 95% CI: −1.36; −0.23). Moreover, no significant differences were shown for DN alone compared to DN + PT (MD: 1.785; 95% CI: −1.807; 5.376) and DN compared to other interventions (MD: 1.922; 95% CI: −2.837; 6.682). However, heterogeneity was high for all the studies (*I*^2^ = 80.08%).

Heterogeneity was moderate for studies in which DN was compared to other interventions (*I*^2^ = 58.29%). Moreover, a low heterogeneity was found for subgroups DN vs DN + PT (*I*^2^ = 0%) and for subgroups DN + PT vs PT (*I*^2^ = 0%).

**Figure 12:**
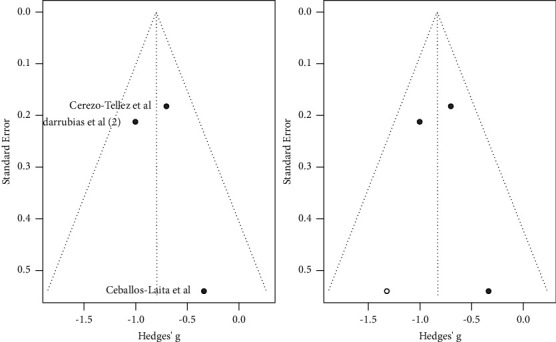
Function subgroup analysis by intervention (DN + PT vs PT). Funnels plot.

Finally, as shown in Figure 12, all studies followed a symmetric distribution. Nevertheless, the studies included in the analysis probably had publication bias or were simply devoted to the analysis. Moreover, the effect sizes of two studies were high.

## 8. Discussion

The objective of this meta-analysis was to compare the effectiveness of DN on pain and function, combined or alone, in patients with chronic neck pain at short-, mid-, and long-term intervals. We found high to moderate evidence suggesting a positive effect of including DN into physical therapy treatment for improving pain intensity and functional disability at short term when compared with other techniques such as US, MT, DN + PT, or stretching alone. In addition, this meta-analysis showed that DN alone improved pain intensity and functional capacity at mid and long term but there were not better results if DN was compared to stretching, MT and exercise at mid and long term. A recent meta-analysis from Fernández-De-Las-Peñas et al. [37] showed the effectiveness of DN techniques to treat neck pain, regardless of chronicity, when compared to other techniques. However, our meta-analysis also showed this effect in the case of chronic neck pain, providing evidence about its effectiveness depending on age and sex.

Liu et al. [38] researched the effects of DN alone at short- and mid-term intervals, showing that wet needling was more effective than DN. However, our study showed differences supporting positive changes at pain intensity and function when performing DN. The presence of studies showing that wet needling (WN) was more effective than DN makes WN an alternative to DN to be considered in future studies. Moderate to low evidence was obtained about the efficacy of DN for pain and function, according to Navarro-Santana et al. [39]. However, positive results on these variables after DN techniques were observed at short term (2–12 weeks) in our meta-analysis. Our meta-analysis showed improvements in pain and function, in contrast with Liu et al. [38], who only showed improvements in pain intensity. The samples included in our meta-analysis differ greatly from that of Liu et al. [38], which analysed a sample of poststroke subjects. The sample from our study was joined by subjects with chronic neck pain, providing updated evidence of DN in chronic neck pain.

Authors such as Navarro-Santana et al. [39] and Cagnie et al. [40] only reported short- and mid-term effects with DN, whereas our meta-analysis also showed that DN was effective in the long term for pain and function. In addition, Navarro-Santana et al. [39] only established a comparison between isolated DN versus other therapies, while our study showed the comparison of DN (alone or combined with other techniques) versus other therapies. Finally, we would like to highlight the homogeneity of the professional performing DN in our study given that 100% of the cases were performed by physiotherapists, in contrast to the 50% reported by Navarro-Santana et al. [39].

Similarly to Liu et al. [38], our study verified that DN is effective for neck pain, at least at short term, for patients with chronic neck pain. Further studies are required to extrapolate these positive effects in the mid and long term. Unlike Liu et al., our study showed that combining DN with other techniques showed significant effects for treating pain and dysfunction in patients with chronic neck pain. These findings could be related to practical guideline recommendations [41] for multimodal treatment for patients with chronic pain.

All studies included in our meta-analysis showed long-lasting effects of DN, either alone or combined with other therapies. This is contrary to Cagnie et al. [40], who found this finding in only one of the studies [33]. Moreover, most of the studies reviewed in our meta-analysis had a dosage of 1 to 3 sessions of DN for 2 weeks (at most). However, Cagnie et al. [40] applied 1 to 6 sessions of DN for 10 weeks. This dosage variability demonstrates that the exact dosage needs to be further studied to obtain benefits with DN.

Our results should be analysed, taking into consideration the strengths and weaknesses of this meta-analysis. The strengths include a thorough and updated search of the scientific literature on the subject that it has been carried out with methodological rigour, that it covers randomised clinical trials of high methodological quality, and that the muscles involved are detailed in almost all the studies. Among the limitations, the DN procedure was not described homogenously throughout the studies, and patient blinding was not assessed and/or achieved in most of the studies, being one of the most common biases in physiotherapy studies. DN should be applied with a diagnosis of MTPs. However, some of the studies analysed in our meta-analysis did not consider the diagnosis of a hyperirritable area in a skeletal muscle associated with a hypersensitive palpable nodule located in a taut band of muscle fibre [7] in their inclusion criteria. It would be interesting to take this diagnosis into account for future studies of chronic neck pain patients. Moreover, the choice of studies published after 2010 as selection criteria may have influenced the inclusion of studies. This bias was mitigated by a previous search of all possible studies for inclusion, noting that those published before 2010 were not directly related to chronic neck pain. In addition, previous systematic reviews published on dry needling and neck pain included these studies. Also, the results in heterogeneity may be affected by the low number of studies, having to interpret the results carefully.

For future research, there is a lack of research about the effectiveness of DN in chronic neck pain at long term. Likewise, some standardised protocols are necessary, which may include the parameters of applying the DN technique for chronic neck pain, the definition of dosage criteria based on the type of patient, and the establishment of an adequate sham DN technique. In addition, it may be interesting to observe the effects between performing superficial and deep DN.

## 9. Conclusion

Our meta-analysis supports the use of dry needling to improve pain and functional capacity in patients with chronic neck pain at short- and mid-term intervals. However, at long term, the number of studies were less numerous, and their results are contradictory. Positive effects in favour of dry needling versus other therapies were found in the studies including patients with a mean age over 40 years in terms of pain, but the same did not occur for the population below 40 years, in which no positive effects were observed. In relation to the interventions, dry needling combined with physical therapy showed to be effective to decrease pain, whereas isolated dry needling did not demonstrate significant improvements in the analysed studies.

Moreover, dry needling did not show to have a different effectiveness to improve function depending on the sex and age. Finally, as for pain, dry needling combined with physical therapy was the therapy that showed the most benefits in function in the analysed studies.

## Figures and Tables

**Table 1:** Studies characteristics.

Study	*N* (age)	Intervention	Muscles involves	Dosage and follow-up	Outcomes	Results
Campa-Moran et al. [23]	*N*: 36 (18−75 y)	G1 (n: 12): DN + stretch G2 (n: 12): MT G3 (n: 12): soft tissue treatment	Upper trapezius bilateral Levator scapulae bilateral	Tt: 2 ss (break of 48 h) DN: at least 3 LTR (2 min each point) Needle: 0.26 × 25 mm Follow-up: baseline, post 1^st^ ss, post 2^nd^ ss, and 1 week	VAS NDI (B, 1 wk) PPT ROM PCS (B, 1 wk) AE	G1: improved NDI, VAS, and flexion at 1 wk G2: improved in all outcomes and follow-ups G3: not improved G2 > G1 fx-Ext ROM + PPT (C5-C6) G2 > G3 PPT + ROM AE: no

Ceballos-Laita et al. [24]	*N*: 21 (30−65 y)	G1 (n: 7): DN + control G2 (n: 7): DNs + control G3 (n: 7): control (TENS + US)	Active MTrPs (at most 3 pts that reproduce symptoms)	G1/G2: G3 + 1 ss/wk (2 wk) G3: 5 ss/wk (2 wk)-15 min TENS + 5 min US + 10 education DN: reach LTR Needle: 0.25 × 40 mm Follow-up: baseline and postintervention	VAS NDI TSK PCS HADS GROC	G1 improved in all outcomes except HADS G1 > G2 and G3 for VAS, NDI and PCS G1 ⟶ 71.4% “great deal better” G2 + G3 ⟶ “moderately better” (71.4%; 42.9%)

Cerezo-Tellez et al. [25]	*N*: 138 (>18 y)	G1 (n: 64): DN + stretch G2 (n: 64): stretch	Trapezius Levator scapulae Splenius cervicis Multifidi	Tt: 2 ss/wk (2 wk) DN: 4-5 LTR Needle: 0.32 × 40 mm Follow-up: baseline, 1 wk, 3 wk, 1 m, 2 m, 4 m, 7 m	VAS NDI PPT ROM Strength AE	G1 and G2 improved in all outcomes G1 > G2 in all outcomes and follow-ups| AE: no

Gallego-Sendarrubias et al. [26]	*N*: 50 (18–60)	G1 (n:25): DN G2 (n:25): DNs	Upper trapezius	Tt: 1 ss DN: 3-4 LTR Needle: x Follow-up: baseline, 1 d, 1 wk	VAS PPT GROC AE	G1 > G2 in all outcomes G1 ⟶ GROC ≧+5 AE: mild

Gallego-Sendarrubias et al. [27]	*N*: 101 (18−55 y)	G1(n: 47): DN + MT G2 (n: 53): DNs + MT	Upper + lower trapezius Levator scapulae	Tt: 1 ss/wk (2 wk) 55 min (5 min DN + 50 MT) DN: 10s up & down Needle: 0.32 × 40 mm Follow-up: baseline, postintervention, and 1 m	NPRS NDI (*B* + 1 m) PPT ROM AE	G1 and G2 improved NPRS, NDI and PPT all follow-ups G1 > G2 in all outcomes AE: no

Garcia-De-Miguel et al. [28]	*N*: 44 (>18 y)	G1 (n: 22): DN G2 (n: 22): DN + PENS	Levator scapulae	Tt: 1 ss 55 min (5 min DN + 50 MT) DN: 8–10 needle insertions Needle: 0.25 × 25 mm Follow-up: baseline, postintervention, 48 h, and 1 wk	VAS NDI (1 wk) PPT ROM Strength	G1 and G2 improved in all outcomes G2 > G1 on NDI and PPT for all follow-ups

Leon-Hernandez et al. [29]	*N*: 62 (18–48 y)	G1 (n: 31): DN G2 (n: 31): DN + PENS	Upper trapezius	Tt: 1 ss DN: 2 LTR Needle: 0.32 × 40 mm Follow-up: baseline, postintervention, 24 h, 48 h, and 72 h	VAS (postDN soreness [24,48, 72 h] & pain [post, 72 h]) NDI (post, 72 h) PPT (post, 72 h) ROM (post, 72 h)	G1 and G2 on pain, soreness, NDI, ROM extension & lateral flexions in all follow-ups G2 > G1 on pain and PPT in postintervention. Not differences between groups for other outcomes

Manafnezhad et al. [30]	*N*: 72 (>18 y)	G1 (n: 36): DN G2 (n: 36): ESWT	Upper trapezius	Tt: 1 ss/wk (3 wk) DN: 1-2 LTRs Needle: 0.32 × 40 mm Follow-up: baseline, 1 wk	NPRS NDI PPT	G1 and G2 improved in all outcomes G1 > G2 in NDI

Navaee et al. [31]	*N*: 40 (18−35 y)	G1 (n: 20): DN G2 (n: 20): stretch	Upper trapezius	Tt: 2 ss/wk (3 wk) DN: until LTRs finished Needle: 0.3 × 50 mm Follow-up: baseline and 1 wk	VAS PPT	G1 and G2 improved in all outcomes and follow-ups G1 > G2

Pecos-Martin et al. [32]	N:72	G1 (n: 36): DN G2 (n: 36): control (1.5 cm of TrP)	Lower trapezius	Tt: 1 ss DN: 8–10 needle insertions Needle: 0.25 × 25 mm Follow-up: baseline, postintervention, 1 wk, and 1 m	VAS NPQ PPT	G1 improved in all outcomes and follow-ups G1 > G2

Stieven et al. [33]	*N*: 44 (18–50 y)	G1(n: 15): DN G2 (n: 14): MR G3 (n: 15): DNs	Upper trapezius	Tt: 1 ss DN: 3 LTR Needle: 0.25 × 30 mm Follow-up: baseline, immediately postintervention, and 10 min after	NPRS PPT NDI (B) FABQ (B) AE	G1 + G2 improved in all outcomes and follow-ups. Not G3 G1/G2 > G3 G1 > G2 AE: no

Stieven et al. [34]	*N*: 116 (18–65 y)	G1 (n: 58): DN + PT G2 (n: 58): PT	Upper and middle trapezius Cervical multifidi Splenius cervicis Levator scapulae	Tt: 4-6 ss/4 wk 40 min DN: 6 LTR Needle: 0.25 × 40 mm Follow-up: baseline, 1 m, 3 m, and 6 m	NPRS NDI GPES PSQI PCS PSEQ AE	G1 > G2 all outcomes at 1 m. Not for 3 m and 6 m AE: mild

Valiente-Castrillo et al. [35]	*N*: 62 (18−65 y)	G1 (n:21): DN G2 (n:21): DN + education G3 (n:20): usual care (electrotherapy)	Upper trapezius Cervical multifidi Splenius cervicis Levator scapulae	G1: 3 ss/wk (2 wk) G2: G1 + 3 ss education G3: 5 ss/wk (2 wk) DN: 5 LTR Needle: 0.32 × 40 mm Follow-up: baseline, postintervention, 1 m, and 3 m	VAS NDI TSK PCS FPQ SOPA PASS-20 AE	G1 and G2 improved et al. follow-ups. G3 only postintervention G1/G2 > G3 for all follow-ups G2 > G1 for postintervention AE: no

Zheng et al. [36]	*N*: 169 (>18 y)	G1 (n: 81): DN (UG) G2 (n: 88): MNS (UG)	Posterior to the articular process of C6	Tt: 1 ss/wk (3 wk) DN: 2-3 insertions Needle: x Follow-up: baseline, 3 m, and 6 m	VAS NDI SF-36 AE	All outcome improved for both groups et al. follow-ups G2 > G1 for all outcomes and follow-ups AE: mild

^*∗*^AE: adverse event; DN: dry needling; DNs: sham dry needling; ESWT: extracorporeal shock wave therapy; FABQ: fear-avoidance beliefs questionnaire; FPQ: fear pain questionnaire; GROC: global rating of change scale; GPES: global perceived effect scale; HADS: hospital anxiety and depression scale; LTR: local twitch response; MT: manual therapy; m: month; MR: myofascial release; NPRS: numeric pain rating scale; NPQ: neck pain questionnaire; NDI: neck disability index; PPT: pressure pain threshold; PCS: pain catastrophizing scale; PSQI: Pittsburgh sleep quality index; PSEQ: pain self-efficacy questionnaire; PASS-20: 20-point pain anxiety symptoms scale; PENS: percutaneous electrical nerve stimulation; PT: physical therapy; ROM: range of motion; SF-36: health status scale; ss: sessions; SOPA: survey of pain attitudes; TSK: Tampa scale of Kinesiophobia; TENS: transcutaneous electrical nerve stimulation; Tt: treatment; TrP: trigger point; US: ultrasound; VAS: visual analogue scale; wk: week; y: years.

**Table 2:** PEDro scale.

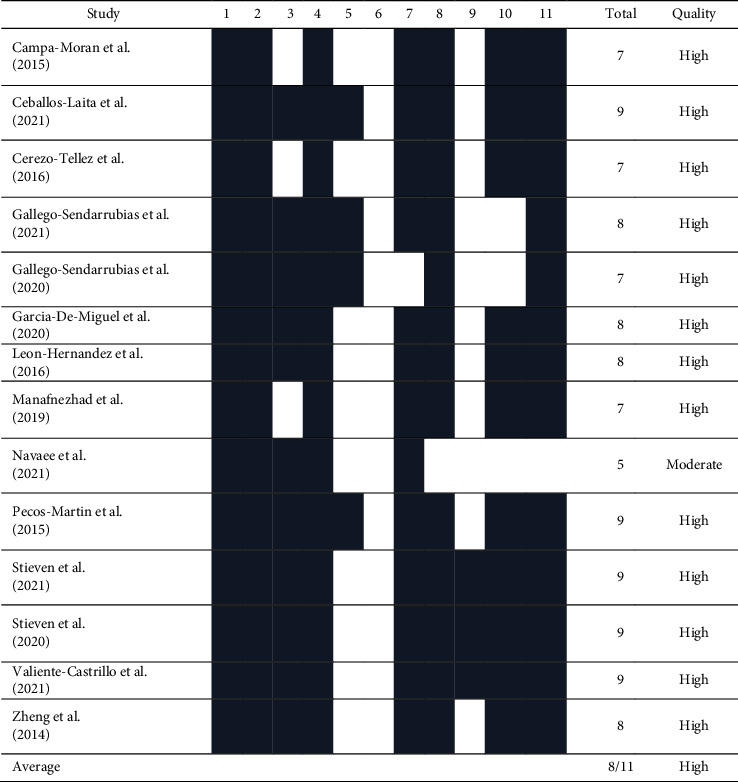

## Data Availability

Data of the systematic review and meta-analysis are available from the corresponding author upon request.

## Supplementary Materials

Supplementary Materials Figure S1. PRISMA 2020 abstracts checklist. Figure S2. PRISMA 2020 checklist. Figure S3. PICO search strategy followed in different electronic document databases. Figure S4. Gosh plot of age in pain outcome. Figure S5. Gosh plot of intervention in pain outcome. Figure S6. Gosh plot of intervention in function outcome.
